# Concrete surface crack detection with the improved pre-extraction and the second percolation processing methods

**DOI:** 10.1371/journal.pone.0201109

**Published:** 2018-07-26

**Authors:** Zhong Qu, Fang-Rong Ju, Yang Guo, Ling Bai, Kuo Chen

**Affiliations:** 1 School of Software Engineering, Chongqing University of Posts and Telecommunications, Chongqing, People’s Republic of China; 2 College of Computer Science and Technology, Chongqing University of Posts and Telecommunications, Chongqing, People’s Republic of China; Istituto Italiano di Tecnologia Center for Micro BioRobotics, ITALY

## Abstract

Monitoring the instantaneous and changing concrete surface condition is paramount to cost-effectively managing tunnel assets. In practice, detecting cracks efficiently and accurately is a very challenging task due to concrete blebs, stains, and illumination over the concrete surface. Unclear and tiny cracks cannot be detected effectively. In this paper, we proposed an ultra-efficient crack detection algorithm (CrackHHP) and an improved pre-extraction and second percolation process based on the percolation model to address these issues. Our contributions are shown as follows: 1) apply the overlapping grids and weight-based, redefined pixel value to obtain the candidate dark pixel image while preserving the cracks. 2) introduce the second percolation processing to generate a high-accuracy crack detection algorithm, which can connect the tiny fractures and detect the tiny cracks. 3) construct a high-efficiency and high-accuracy crack detection algorithm combining the improved pre-extraction and the second percolation process. The experimental results demonstrate that CrackHHP can significantly improve the efficiency and accuracy of crack detection.

## 1. Introduction

Concrete surface crack detection is very important for the maintenance of concrete structures [1]. Traditional manual inspection approaches are very subjective, time-consuming, and labor-intensive. By employing the industrial CCD camera to catch the crack images, the accurate automatic measurements by digital image processing can overcome the shortage of manual inspection to achieve effective preventative maintenance [2–3].

Due to the topology complexity of cracks and noise diversity over the concrete surface crack images, detecting the tiny cracks accurately in different environments is a challenging task [4–5]. The most basic methods are based on a threshold segmentation operation [6]. In the [7], six different segmentation algorithms that were used to evaluate the performance of image segmentation in automatic image distress detection and classification. Full automation has remained a challenge [8–9].

Based on the ground, penetrating radar [10], laser [11] or remote sensing system [12] were proposed to evaluate the road quality. These methods can rapidly collect information over wide areas and attain quasi-continuous crack detection results and the depth of cracks [13]. But configuration requirements need to be designed seriously for different situations. For nondestructive evaluation of surface degradations, computer-based digital image-processing approaches seem more promising in recent research results [14] [15].

Saliency detection can distinguish target cracks in contrast to the background effectively. Existing methods using texture smoothing [15] and local statistical features [16], and global features [17] can perform well on saliency object detection in MSRA-1000 dataset, but the result of crack completeness and continuity is not well. Automatic road crack detection was used to detect and analyze multiple cracks [18] [19].

With the existence of wide crack databases, machine learning based methods have been used to detect cracks. Yong Shi et al. [20] proposed a crack descriptor based on the random structured forests [21] to discern cracks from noises effectively, but it does not perform well while dealing with cracks with connectivity edge noises. The convolution neural network(CNN) based methods [22][23] favored the local crack patches using Deep Learning. They were especially strong at detecting thin cracks under lighting conditions that make detection difficult when using traditional methods. However, they relied on the training dataset that must be tuned in the same concrete condition, and a CNN-implemented method required a large amount of training data to train a robust classifier.

In addition, some researchers enhance the continuity of the existing methods from the global view. Dijkstra’s algorithm was used to the find the best selection of minimal paths [24]. Minimum spanning trees [25] were used to describe the possible connections of sampled crack seeds. These methods provided robust and precise results in a wide range of situation, but they may perceive a crack that does not exist [20].

T. Yamaguchi et al. [26] used scalable local windows to percolate different shapes and positions cracks. But all pixels must be percolated, which costs much computation time. To reduce the computation time of crack detection based on percolation model, Qu et al. in [27] added a termination procedure and a midway skip procedure into the percolation process to improve the efficiency of crack detection. In [28], they used the small grids to accelerate crack detection.

**Fig 1:**
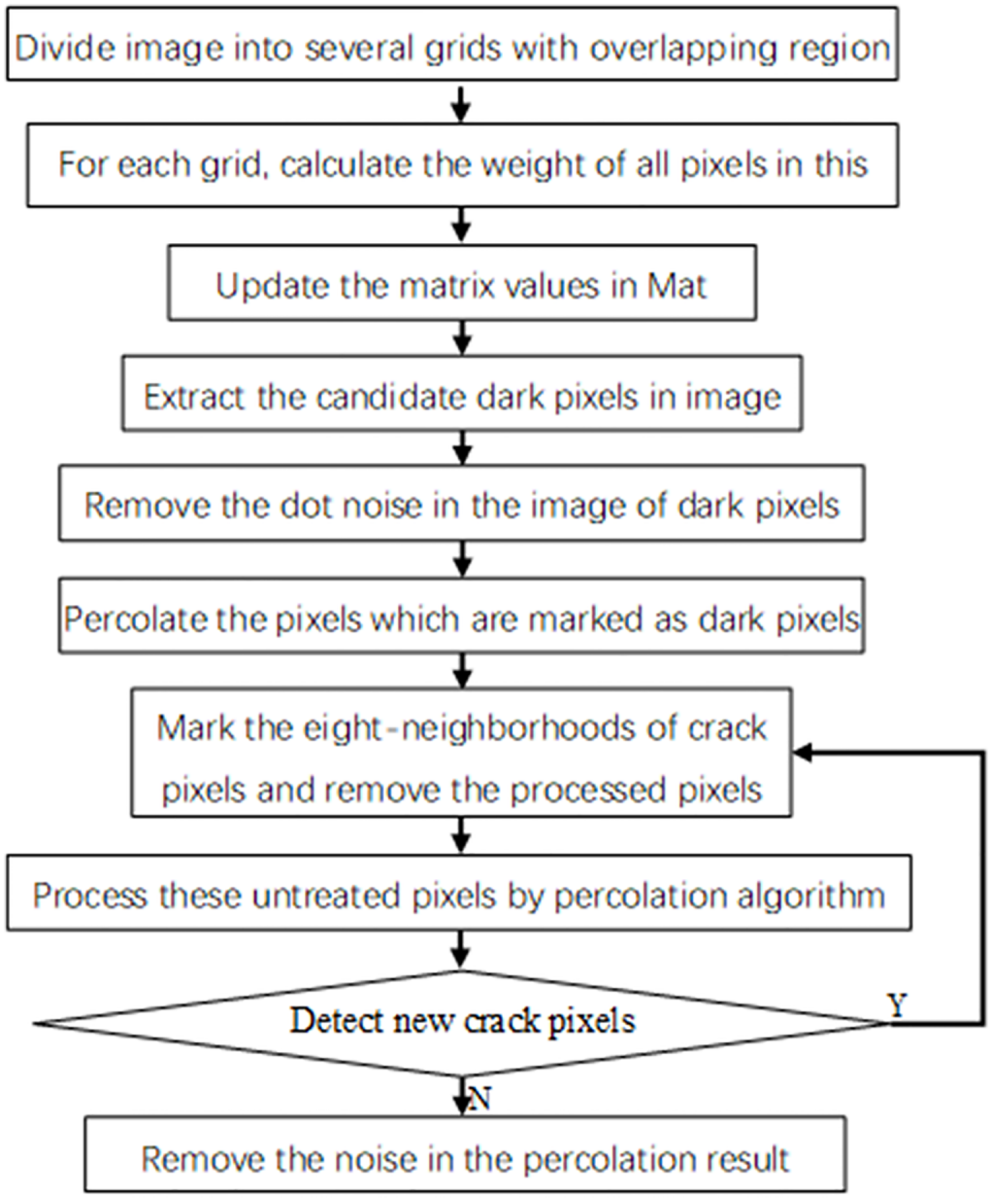
Flow chart of CrackHHP.

In this paper, we proposed a crack detection algorithm CrackHHP. As shown by the flow chart in Fig 1. First, each pixel in the grid is adaptively set a different weight according to pixel brightness. Then, we extract the candidate dark pixels according to the weight of each pixel, remove dot noise, and percolate dark pixels. Finally, we percolate the neighborhood pixels of dark pixels to connect the tiny fractures.

## 2. Existing percolation algorithm

The traditional percolation algorithm needs to percolate each pixel in the image which caused a computational burden. In [28], the author divided the whole image into several small grids before the percolation process to keep the dark pixels and remove brighter background pixels. But a lot of noise is made nearby the grid lines, and a crack in a high luminance region cannot be detected.

**Fig 2:**
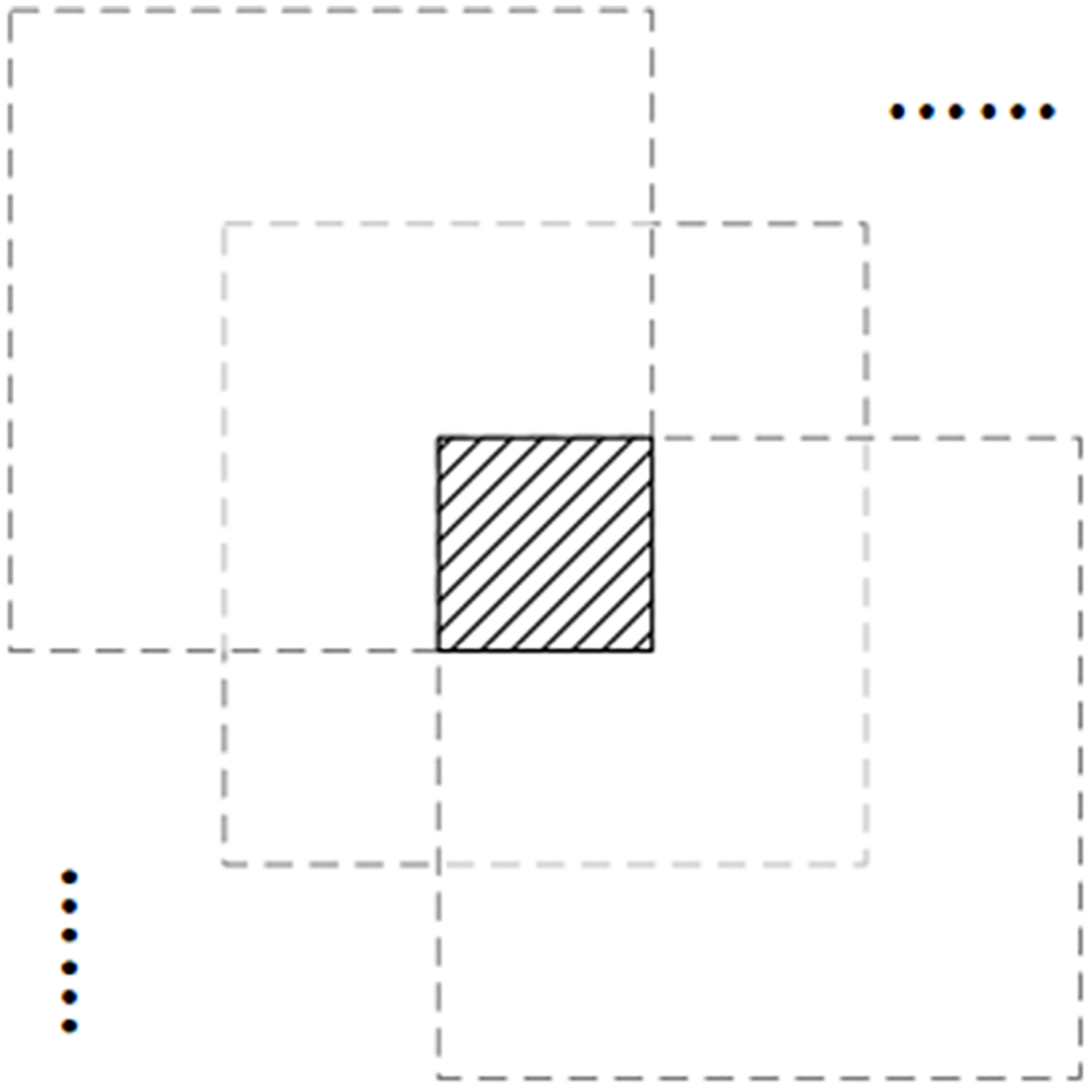
Divided image into several small grids with the overlapping regions.

In this paper, we use overlapping grids instead of small grids. Grids sketched with the overlapping region as shown in Fig 2. The overlap time *C* is the time of grid overlapping in horizontal or vertical direction, which means each pixel will be included in *C*^2^ grids. According to the characteristics of gray-scale, counting the number of extraction times for a given pixel is used to label a candidate dark pixel. The threshold value *T* is defined to judge whether the pixel will act as the dark pixel by comparing the extraction time. If *extract times* ≥ *T*, mark the pixels as the dark pixels for the whole image.

The accelerated crack detection algorithm based on the pre-extraction method and percolation model is described as follows:

(i) Divide image with overlapping grids.
(ii) Sort each grid pixels in ascending order of gray-scale and choose the top *Pct* percent pixels *D*_*N*_.
(iii) When *extract times* ≥ *T*, mark the pixels as the dark pixels of whole image.
(iv) Remove the dot noise in pre-extraction result image with de-noising algorithm based on percolation model [27].
(v) Percolate the pixels which are marked as dark pixels and transform the result to binarization image.

## 3. The high-efficiency and high-accuracy algorithm

### 3.1 The high-efficiency method of percolation algorithm

To further improve the efficiency of crack detection, an improved pre-extraction method based on redefine pixel weight was proposed. In this study, the author found that directly calculating the weight of each pixel with the extreme and average value in each grid is not comprehensive. The light and tiny cracks have an important influence on the accuracy of the experimental result.

Therefore, we consider the effect of brighter pixels and introduce the parameters mean value *L*_*Avg*_ and minimum mean value *L*_*MinAvg*_. *L*_*Avg*_ is defined as the average value in *L*_*p*_. *p* is used to present the pixel in each grid. *L*_*p*_ is the pixels set after removing brighter pixels. *n* is the number of pixels in *L*_*p*_. *I*_*p*_ is the brightness of the pixel *p*. *L*_*Avg*_ is given as follows:

$$L_{Avg}=1/n\sum I_{p},I_{p}\in L_{p}$$ (1)

To avoid the drawback that individual background noise pixel brightness is lower than the crack pixel, *L*_*MinAvg*_ is used to instead of the minimum value in *L*_*p*_. The minimum mean value *L*_*MinAvg*_ is the average value of *L*_*p*2_. *L*_*p*2_ is defined as the multiple front pixels after sorting in ascending order in the grid. *N* is the pixels number in *L*_*p*2_. *L*_*MinAvg*_ is calculated as follows:

$$L_{MinAvg}=1/N\sum I_{p},I_{p}\in L_{p2}$$ (2)

**Fig 3:**
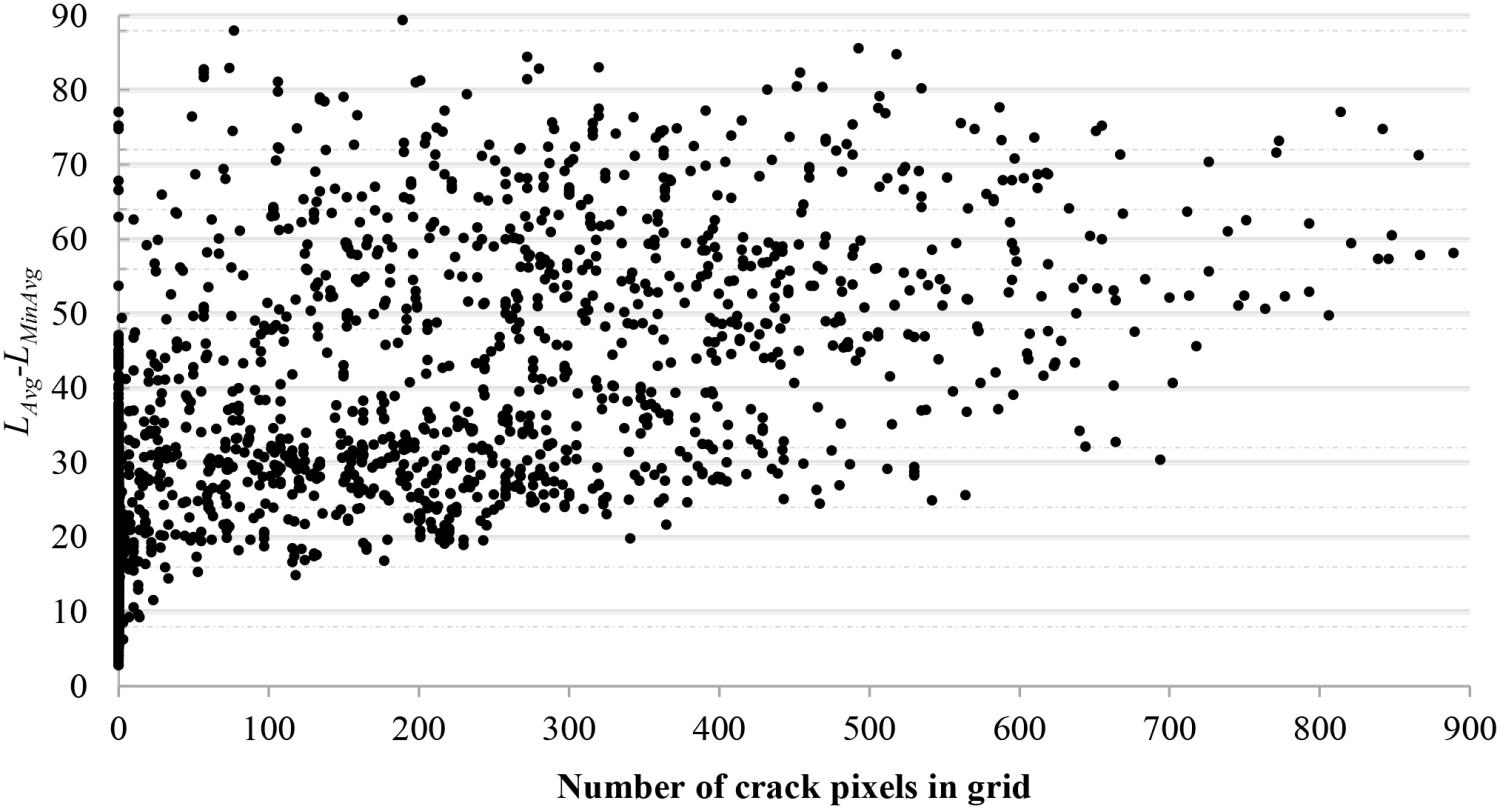
Relationship between crack pixels and *L*_*Avg*_ − *L*_*MinAvg*_.

It can be seen from Fig 3, the value of *L*_*Avg*_ − *L*_*MinAvg*_ is sensitive to the crack pixels in each grid. With the increase of *L*_*Avg*_ − *L*_*MinAvg*_, there are more crack pixels in the grid. We can roughly determine whether the grid contains the crack according to the value of *L*_*Avg*_ − *L*_*MinAvg*_. In general, the greater value of *L*_*Avg*_ − *L*_*MinAvg*_ is, the higher possibility of grids containing cracks is, and more candidate dark pixels can be extracted. When the value of *L*_*Avg*_ − *L*_*MinAvg*_ is small, few, if any, candidate dark pixels or can be extracted.

The specific high-efficiency algorithm with the improved pre-extraction process as follows:

(i) Define a matrix *Mat*(*i*,*j*)(*i*∈[0,*height*−1], j∈[0,*width*−1]) with size of import image *height* and *width*. And initialize the matrix as zero.
(ii) Divide image with overlapping grids.
(iii) Sort each grid pixels *num* in ascending order of gray-scale.
(iv) Calculate *L*_*Avg*_ and *L*_*MinAvg*_.
(v) If *L*_*Avg*_ − *L*_*MinAvg*_ < *T*_1_, set the weight *W* of all the pixels in the grid as -0.5; if *T*_1_ ≤ *L*_*Avg*_ − *L*_*MinAvg*_ < *T*_2_, set the weight *W* of all the pixels in the grid as 0.
(vi) If *T*_2_ ≤ *L*_*Avg*_ − *L*_*MinAvg*_ < *T*_3_, set the extracting proportion *P*_1_ = 0.5+(*L*_*Avg*_ − *L*_*MinAvg*_−*T*_2_)/*T*_3_; if *T*_3_ ≤ *L*_*Avg*_ − *L*_*MinAvg*_, set the extracting proportion *P*_2_ = 1.
(vii) Choose the front part pixels *D*_*N*_ of each grid pixels *num*. *D*_*N*_ is *num* × *Pct* × *P*_1_.
(viii) Set weight *W* to each pixel in *D*_*N*_. *rank*(*D*_*N*_) means the position of pixels after sorting by ascending order in the grid, *W* is calculated as follows:
  $$W=1-\frac{rank(D_{N})}{num}$$ (3)
(ix) *Mat*(i,j) is updated as follows:
  Mat(i,j)=Mat(i,j)+W (4)
(x) Sort the weight *W* by descending order, choose the value of front part pixels after sorting and the number is *num* × *R*, *R* is the ratio of extracted dark pixels. Generally, the proportions of the crack pixels in each image are less than 5%, we set *R* = 0.05. Then remove the pixels when *W* < *W*_*T*_ and mark the rest pixels in dark pixels image. *W*_*T*_ is related to the overlap time *C*, we set *W*_*T*_ = 4.5. *R* and *W*_*T*_ are empirical value.
(xi) Percolate the dark pixels and transform the result to binarization image.
(xii) Remove the noise in percolation result image.

**Fig 4:**
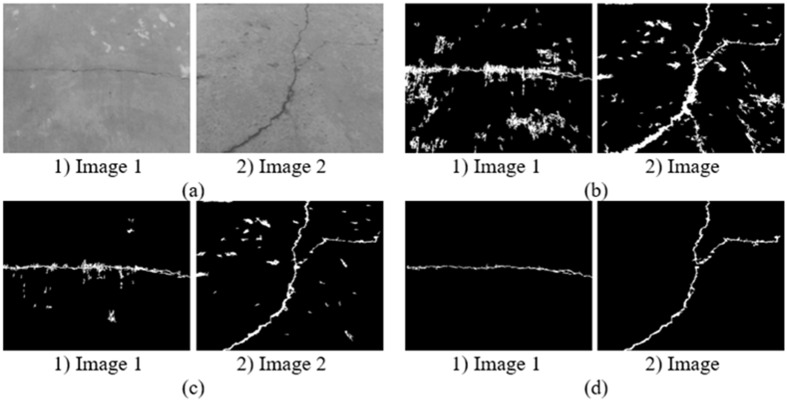
Experimental results of high-efficiency algorithm. (a) Original images. (b) Previous pre-extraction images in Ref. [28]. (c) Improved pre-extraction images. (d) Percolation results of the high-efficiency algorithm.

Results of high-efficiency algorithm are shown in Fig 4. It can be seen intuitively that the improved pre-extraction method significantly reduced the number of dark pixels, in addition, the accuracy of the high-efficiency algorithm is acceptable. For the percolation algorithm, the less extracted dark pixels are, the more quickly the percolation process is. In this way, we can significantly improve the efficiency of the percolation algorithm.

### 3.2 The high-accuracy method of percolation algorithm

In the study, we found that some unclear and tiny crack pixels by the influence of noise are not extracted as dark pixels during the pre-extraction process. There are two reasons. First, under the influence of complex environment, the brightness of some crack pixels is similar to the background. The pre-extraction process only considers the feature of gray level, which cause unclear and tiny cracks cannot be detected. Second, some dot real crack pixels were removed in the de-noising process. In view of the above two points, we considered the shape feature and connectivity characteristic of crack to detect the unclear cracks and fill the tiny fracture in the high-accuracy method which was used to percolate the neighborhood crack pixels again after the first percolation.

The high-accuracy percolation algorithm is as follows, three steps are added after step (viii) in Sec. 2 the crack detection algorithm:

(i′) Mark the eight-neighbourhoods of crack pixels and remove the processed pixels.
(ii′) Process these untreated pixels.
(iii′) If there are new crack pixels, mark the new eight-neighbourhoods crack pixels and remove the processed pixels. Otherwise, the percolation process is terminated.

**Fig 5:**
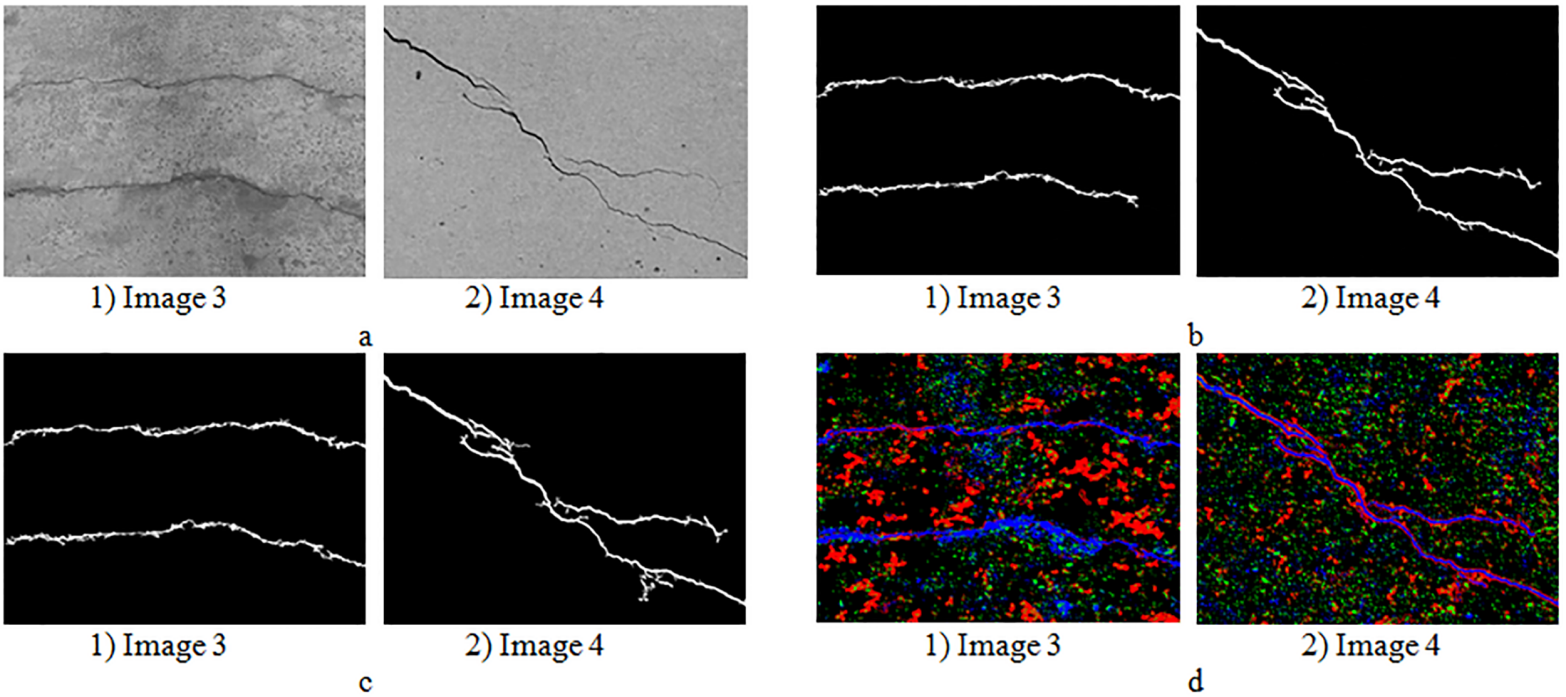
Experimental results of the high-accuracy algorithm. (a) Original images. (b) Results of the previous percolation algorithm. (c) Results of the high-accuracy algorithm. (d) Experimental results analysis images (Blue: the accelerated pixels of the previous pre-extraction method; Green: the pixels of the first percolation; Red: the pixels of the second percolation; Black: without being percolated pixels).

Results of the high-accuracy algorithm compared with previous percolation algorithm are shown in Fig 5. From Fig 5(b) and 5(c), the high-accuracy algorithm can detect more tiny cracks and reduce fractures. Fig 5(d) analyses the percolated pixels in two percolation process. It can be seen that the second percolation process (red pixels) focused on the edge and fractures of cracks, which can detect the tiny cracks and fill up the fractures.

### 3.3 The combination algorithm

**Fig 6:**
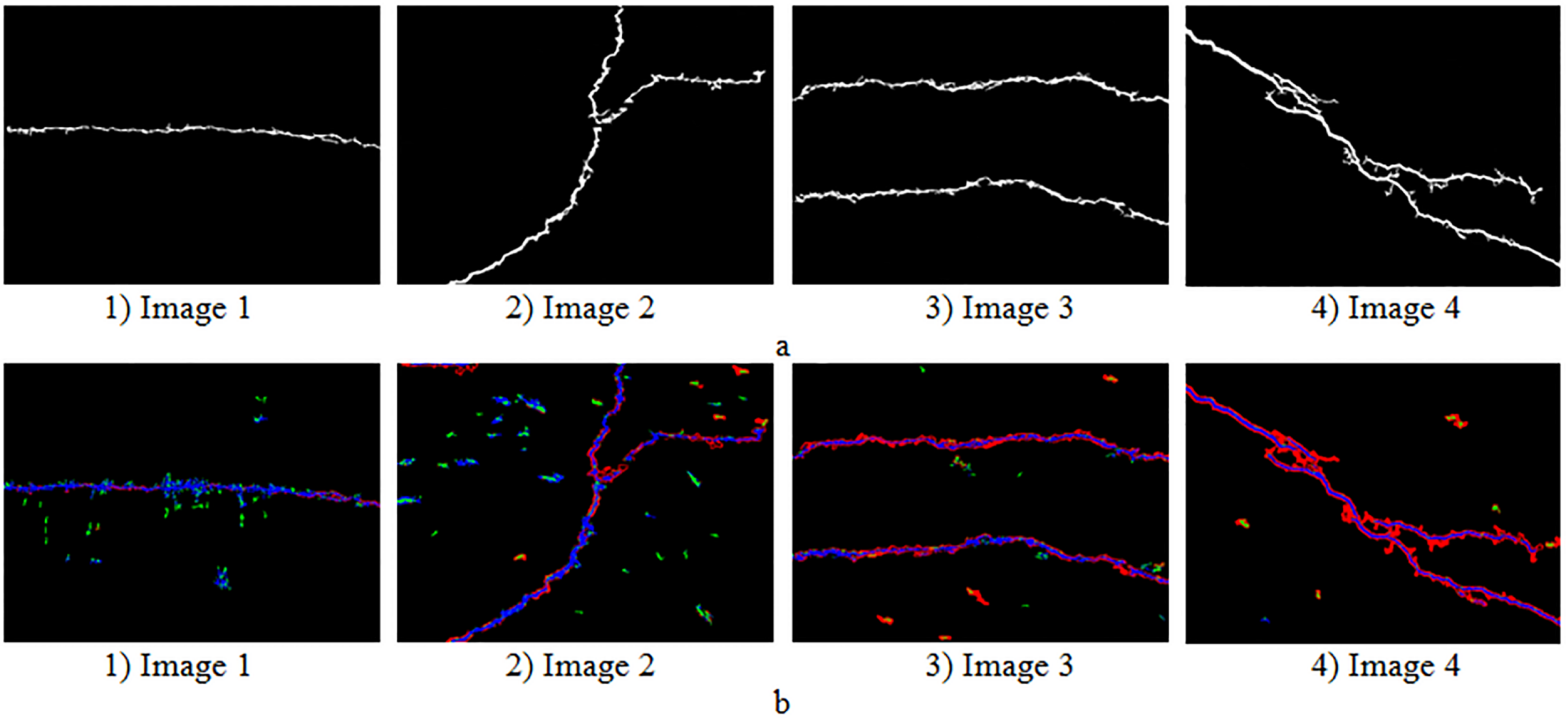
Experimental results of combination algorithm. (a) Percolation results of the combination algorithm. (b) Experimental results analysis images (Blue: the accelerated pixels of improved pre-extraction method; Green: the pixels of the first percolation; Red: the pixels of the second percolation; Black: without being percolated pixels).

The combination algorithm which integrated the pre-extraction method in Sec. 3.1 and the second percolation method in Sec. 3.2 is proposed. First, extract the crack with the high-efficiency algorithm. Then, replace the percolation process in the high-efficiency algorithm with the high-accuracy method which can make up for the problem in the precision rate and recall rate caused by the high-efficiency algorithm. Results of CrackHHP are shown in Fig 6.

It can be seen from Fig 6(a) that the combination algorithm can detect more cracks in the image, especially some blurred cracks. In Fig 6(b), the number of pixels which need to be percolated is less than the high-accuracy algorithm significantly (compared with Fig 6(d)). Therefore, the combination algorithm can greatly improve the speed of the percolation process and maintain a high precision.

## 4. The experimental results and analysis

### 4.1 Evaluation of accuracy

In this section, we analyze the performance of the high-efficiency and high-accuracy algorithm. We have showed results on two datasets (S1 Fig). One dataset includes 100 natural environment concrete surface images taken by our team, which can generally reflect urban road concrete surface condition in Chongqing, China. All the images are taken by Canon 5D mark II with aperture of f/4.0, shutter of 1/400, iso of 100 and awb of auto. The image size is 400×300 pixels. Another dataset CFD [20] proposed by Yong Shi et al. that allows for quantitative analysis due to ground truth data. This dataset is composed of 118 images. The image size is 480×320 pixels.

In order to evaluate our algorithm, we compare it with the previous percolation algorithm [28], CrackForest [20], CrackTree [25], and FFA [29]. In quantitative analysis, we use precision rate *P* and recall rate *R* refer to (5) and (6) to evaluate the experimental results,

P=Np/Nt (5)

R=Np/Nr (6)

where *Np* is the number of true crack pixels detected in the percolation process, *Nt* is the number of pixels detected as cracks, *Nr* is the total pixels number of detected cracks in ground truth image.

**Fig 7:**
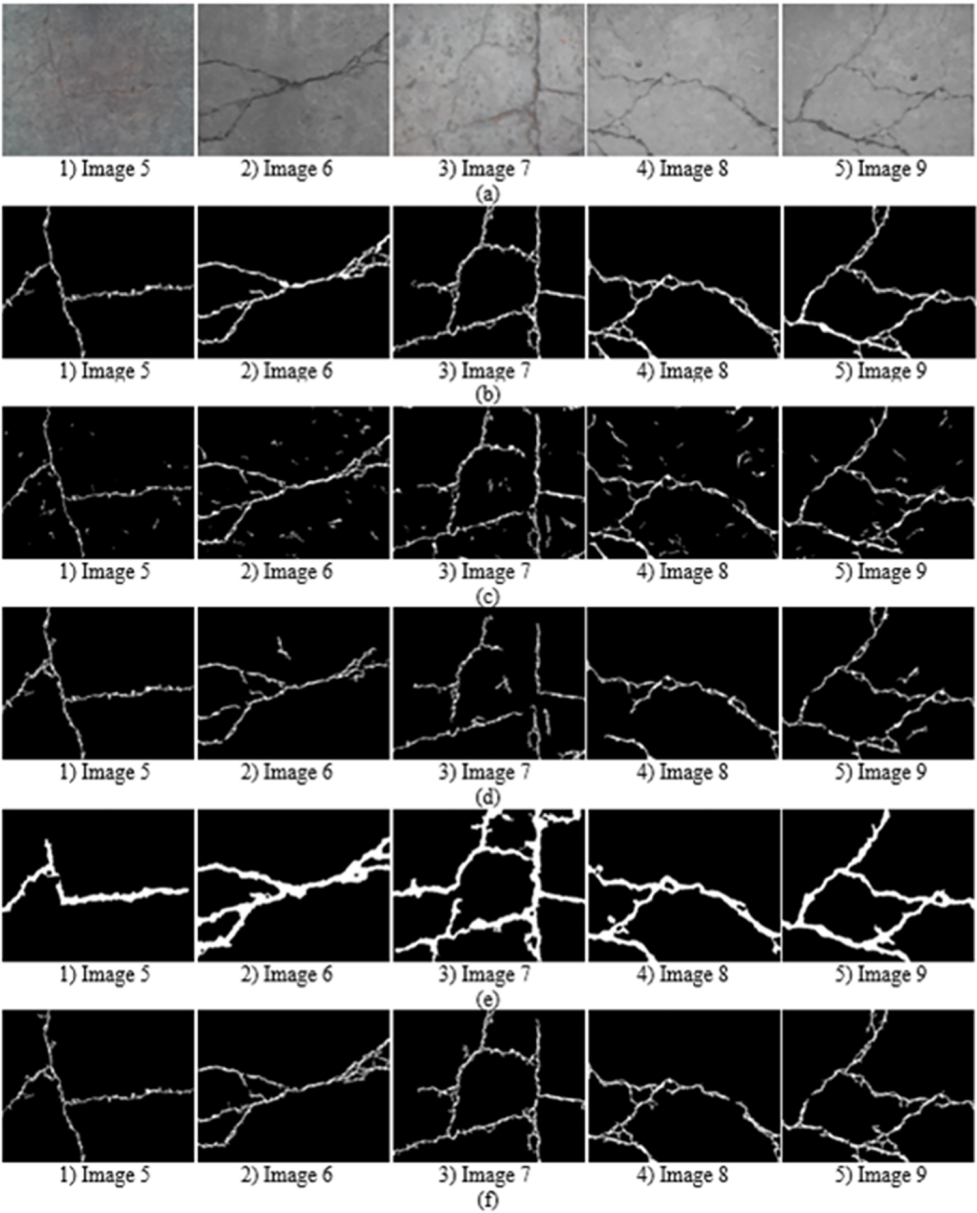
Experimental results with different algorithm. (a)original images. (b)ground truth. (c)the improved pre-extraction images. (d)previous percolation algorithm. (e) CrackForest. (f) CrackHHP.

**Fig 8:**
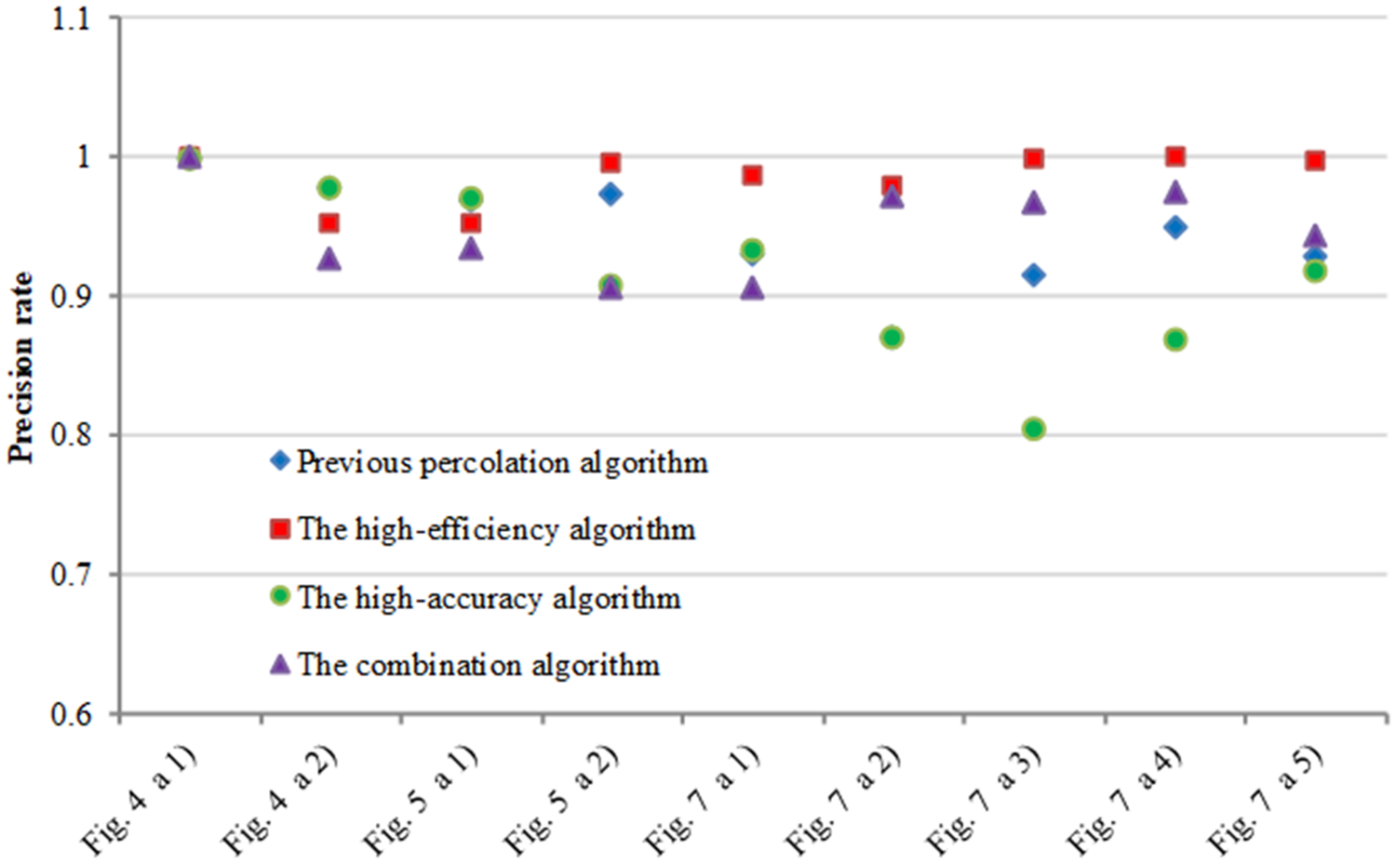
Precision rate comparison.

**Fig 9:**
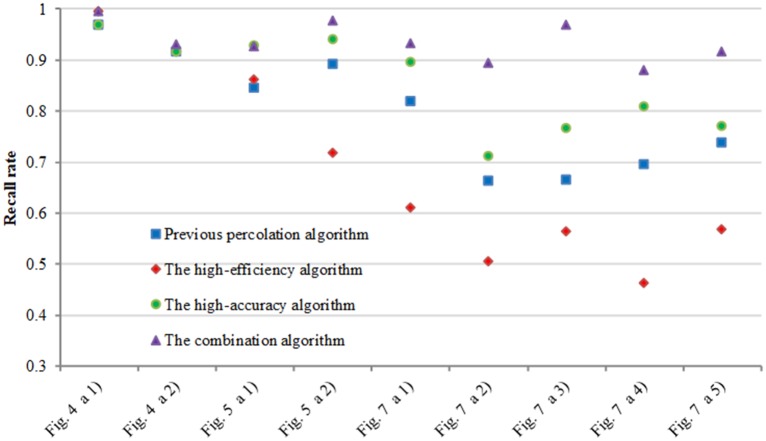
Recall rate comparison.

Fig 7 show the comparison experimental results on the first dataset. We can see these images include complex topology and tiny cracks. The improved pre-extraction method can effectively extract crack dark pixels. The accuracy of the previous percolation algorithm is acceptable. However, it is sensitive to noise and loses partial crack information which has an influence on the precision rate and recall rate. CrackForest can quickly extract and fill the crack edge, but the width of the detected crack is thicker than ground truth, which easily causes inaccurate crack information. The comparison of precision rate and recall rate are shown in Figs 8 and 9 respectively. The precision rate of the high-efficiency algorithm and CrackHHP are higher, which means only small noises are detected as cracks. But, the recall rate of the high-efficiency algorithm is not satisfactory. In general, the higher recall rate means the detection result is closer to the ground truth crack image. CrackHHP performs better than others, which gives both high precision and recall.

**Fig 10:**
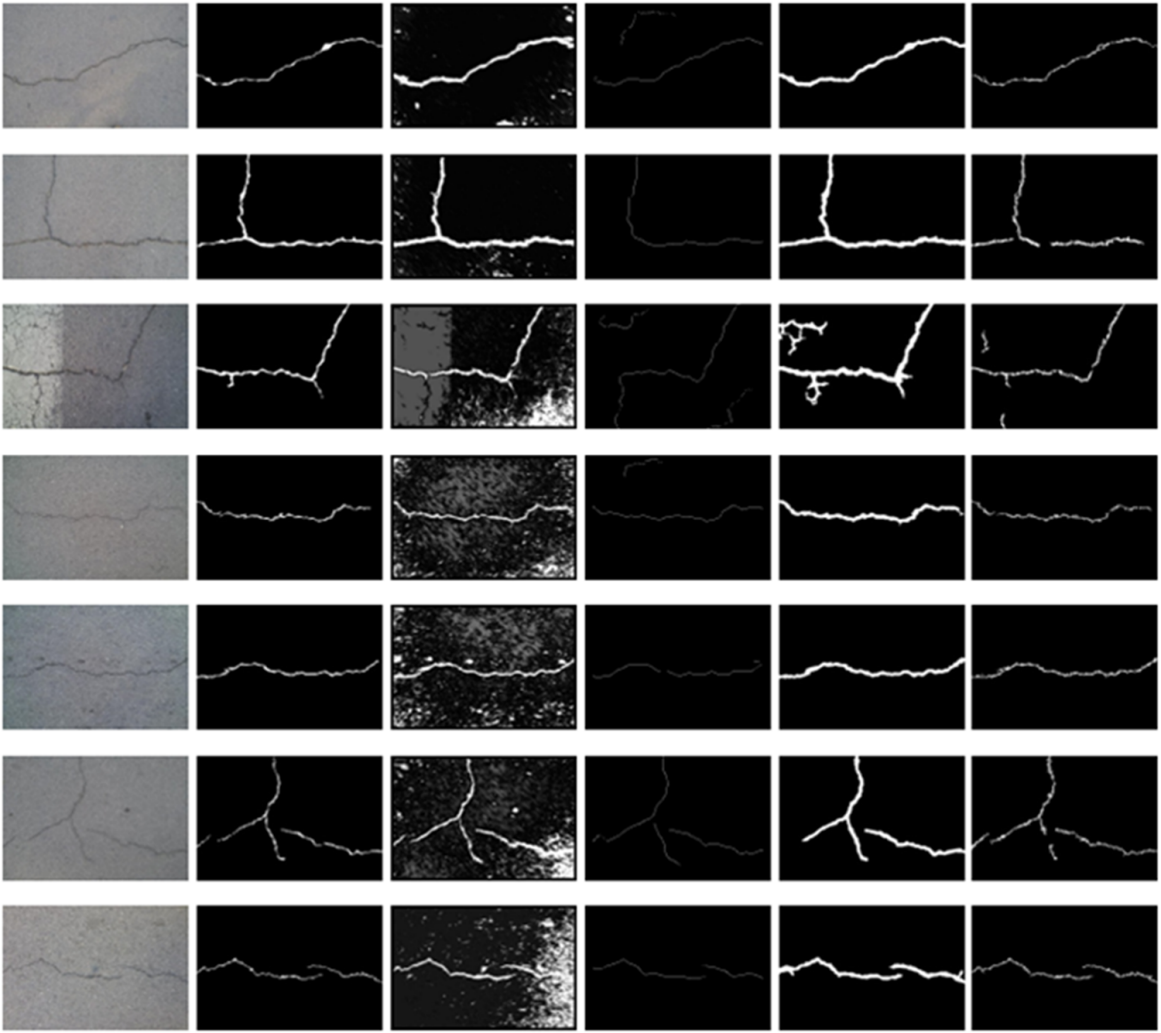
Results of different algorithms on CFD (from left to right: Original image, ground truth, FFA, CrackTree, CrackForest, CrackHHP).

**Fig 11:**
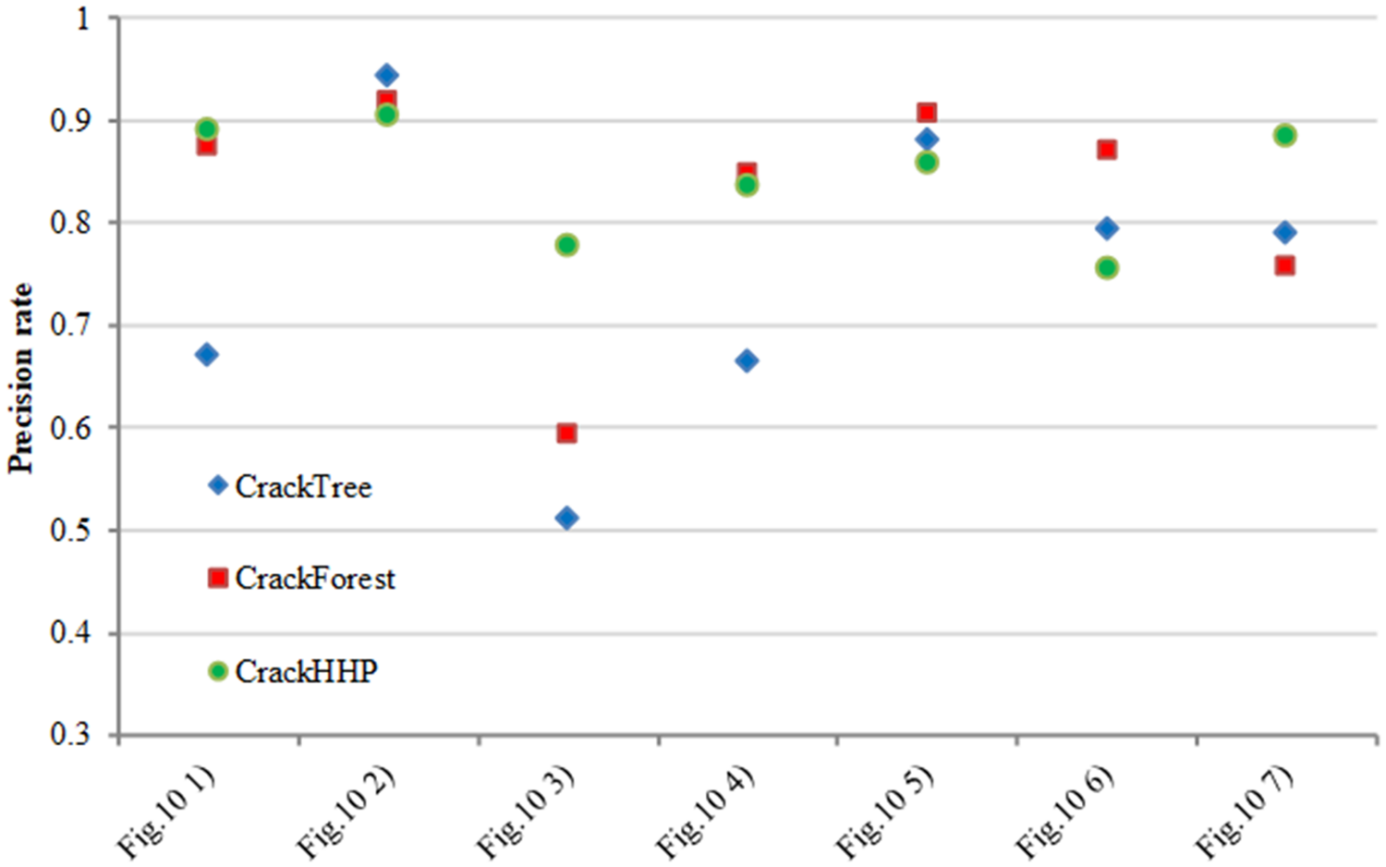
Precision rate comparison.

**Fig 12:**
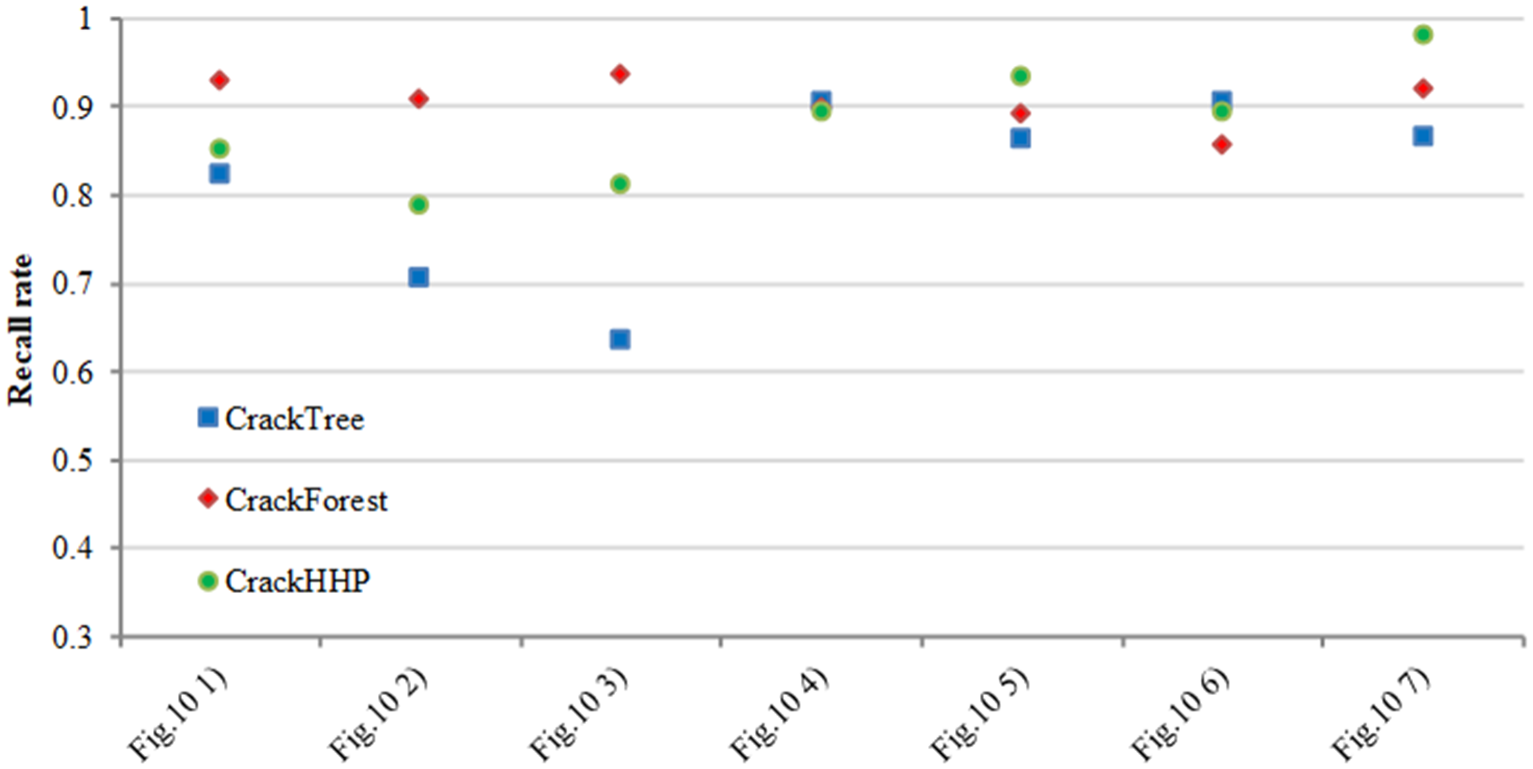
Recall rate comparison.

Fig 10 show the comparison experimental results on the dataset of CFD. We compare four methods on this dataset. The analysis of precision and recall are shown in Figs 11 and 12 respectively. The number Fig 10 1)–10 7) of Figs 11 and 12 corresponding to the first column from top to bottom in Fig 10. FFA can detect a continuous crack, but the abundance of noise near the cracks can cause a small precision level. Although CrackTree can extract effectively the crack skeleton, it may perceive a crack that does not exist. CrackFoerst can quickly and effectively obtain the crack, but it is not good for detecting tiny cracks. CrackHHP performs better than the others. The width of results is closer to the ground truth. Moreover, the precision and recall are both satisfactory.

### 4.2 Evaluation of computation time

The operating environment of the experiments is configured as follows: CPU: AMD A8-5550M 2.20GHz, GPU: AMD Radeon(tm) HD 8570M Graphics, OS: Windows 7, Language: C++, Library: Opencv2.4.3.

In Table 1, time-consuming results of several algorithms are shown. The high-efficiency algorithm is the fastest, but the recall is not good. As for the high-accuracy algorithm, the precision inadequate, but the computational time is less competitive. The efficiency of CrackHHP performs better than other algorithms except the high-efficiency algorithm, which has the highest recall rate. To summarize, the proposed CrackHHP is better on the accuracy and computational time than other algorithms.

**Table 1:** The time-consuming comparison.

Time(s)	Traditional percolation algorithm	Previous percolation algorithm	The high-efficiency algorithm	The high-accuracy algorithm	The combination algorithm
Fig 4 a 1)	27.804	3.853	1.030	4.307	1.050
Fig 4 a 2)	42.811	9.391	3.229	11.116	4.259
Fig 5 a 1)	71.802	16.333	2.075	34.611	3.703
Fig 5 a 2)	60.078	24.632	1.747	31.760	7.213
Fig 7 a 1)	68.589	25.844	2.542	35.892	10.577
Fig 7 a 2)	80.025	17.550	4.820	32.880	14.507
Fig 7 a 3)	105.156	25.256	3.947	81.143	15.256
Fig 7 a 4)	78.930	29.017	5.881	67.658	22.801
Fig 7 a 5)	69.779	22.168	4.118	31.012	18.262
Average	67.219	19.338	3.265	36.709	10.848

Table 2 is the detailed efficiency analysis of CrackHHP proposed in this paper. The percolation process is time consuming, especially the second percolation. The improved pre-extraction and the de-noise take less time and the time consumed is relatively stable.

**Table 2:** The efficiency analysis of CrackHHP.

No.	Improved pre-extraction (s)	Denoise1(s)	Percolation(s)		Denoise2(s)	Total(s)
			First time	Second time		
Fig 4 a 1)	0.499	0.061	0.375	0.077	0.038	1.05
Fig 4 a 2)	0.621	0.085	2.449	1.056	0.048	4.259
Fig 5 a 1)	0.641	0.112	1.248	1.651	0.051	3.703
Fig 5 a 2)	0.653	0.122	0.858	5.504	0.076	7.213
Fig 7 a 1)	0.687	0.24	1.743	7.86	0.047	10.577
Fig 7 a 2)	0.628	0.095	3.978	9.728	0.078	14.507
Fig 7 a 3)	0.694	0.083	3.136	11.281	0.062	15.256
Fig 7 a 4)	0.626	0.082	5.085	16.932	0.076	22.801
Fig 7 a 5)	0.64	0.081	3.338	14.133	0.07	18.262

The experimental results show that our method CrackHHP proposed in this paper can be effectively and fast detect concrete surface crack images.

## 5. Conclusion

In this paper, we proposed a crack detection algorithm, CrackHHP, which can significantly improve the percolation speed by extracting dark pixels. Our second percolation process can detect the tiny cracks and fill up the tiny fractures. Experimental results showed that our method can promise the state-of-the-art accuracy and processing speed. In the future, our method can be developed as a support for human visual control applied to monitor the concrete surface condition of public transportation infrastructure for, such as highways, tunnels, and bridges. In addition, other features of cracks can be integrated into in-depth research.

## Supporting information

S1 Fig The experimental images provided by the author are collected in minimal underlying data set.rar. (RAR)
